# Vaccine Formulation Strategies and Challenges Involved in RNA Delivery for Modulating Biomarkers of Cardiovascular Diseases: A Race from Laboratory to Market

**DOI:** 10.3390/vaccines11020241

**Published:** 2023-01-21

**Authors:** Md. Adil Shaharyar, Rudranil Bhowmik, Fahad A. Al-Abbasi, Shareefa A. AlGhamdi, Amira M. Alghamdi, Arnab Sarkar, Imran Kazmi, Sanmoy Karmakar

**Affiliations:** 1 Bioequivalence Study Centre, Department of Pharmaceutical Technology, Jadavpur University, Kolkata 700032, West Bengal, India; 2 Department of Biochemistry, Faculty of Science, King Abdulaziz University, Jeddah 21589, Saudi Arabia; 3 Experimental Biochemistry Unit, King Fahd Medical Research Center, King Abdulaziz University, Jeddah 21589, Saudi Arabia

**Keywords:** non-coding RNA, Si RNA, vaccine, cardiovascular, miRNA inhibitors

## Abstract

It has been demonstrated that noncoding RNAs have significant physiological and pathological roles. Modulation of noncoding RNAs may offer therapeutic approaches as per recent findings. Small RNAs, mostly long noncoding RNAs, siRNA, and microRNAs make up noncoding RNAs. Inhibiting or promoting protein breakdown by binding to 3’ untranslated regions of target mRNA, microRNAs post-transcriptionally control the pattern of gene expression. Contrarily, long non-coding RNAs perform a wider range of tasks, including serving as molecular scaffolding, decoys, and epigenetic regulators. This article provides instances of long noncoding RNAs and microRNAs that may be a biomarker of CVD (cardiovascular disease). In this paper we highlight various RNA-based vaccine formulation strategies designed to target these biomarkers—that are either currently in the research pipeline or are in the global pharmaceutical market—along with the physiological hurdles that need to be overcome.

## 1. Introduction

Even · though most of the genome is transcribed, only around 1–2% of exons actually code for proteins. The remaining noncoding segments consist of short and long-noncoding RNAs (lncRNAs). The vast majority of short noncoding RNAs are microRNAs (miRNAs). MiRNAs, the vast majority of which range in length from 21 to 23 nucleotides, play crucial roles in many aspects of biology, including cell development, proliferation, and survival [1]. The most recent version of miRBase reports that 1881 human miRNA precursors and 2588 human miRNAs have been uncovered. By binding to complementary 3′-untranslated regions of target mRNAs, they prevent translation or trigger mRNA disintegration. The precursor pri-miRNAs are the starting point from which miRNAs are produced. A 70-nucleotide-long premiRNA is produced when a pri-miRNA is synthesised within the nucleus with the help of the drosha-DGCR8 DGCR (DiGeorge syndrome chromosomal region) complex. Following further processing in the cytoplasm, the mature miRNA, which is close to 22 nucleotides in length, is produced [2]. Since a single miRNA may modify the expression level of so many mRNAs and hence influence so many biological processes in numerous tissues, it may not be surprising that expression levels of miRNAs have been discovered in a number of pathological states, such as cardiovascular or metabolic disorders [3,4]. They consequently emerged as viable therapeutic targets. Existing techniques for altering miRNA performance fall into two categories. Overexpression of a target miRNA can be achieved with the use of miRNA mimics or viral vectors, whereas its inhibition can be achieved with antisense oligonucleotides (ASOs) or genetic knockout mice models. Small noncoding RNAs are less diverse than lncRNAs, which are >200 nucleotides long. So far, investigations have led to the identification of over 60,000 lncRNAs in the human genome [5]. Intergenic areas, intronic segments of protein-coding genes, and even antisense strands belonging to a particular gene are responsible for the formation of lncRNAs. Eventually, lncRNAs are also produced via the reverse splicing of exons, which creates circular RNAs. 

They can serve as a scaffolding for protein complexes, a decoy for molecules under target so as to dampen their activity, or an epigenetic regulator of gene transcription [6,7,8]. As an added bonus, lncRNAs can influence transcription and post-transcriptional gene regulation by interacting with DNA, RNA, or miRNAs through splicing or sponging. Like miRNAs, numerous lncRNAs are dysregulated in a wide variety of diseases. Understanding if lncRNAs can be therapeutically targeted like miRNAs will be beneficial. To induce lncRNA degradation and inhibit their function, gapmers or small interfering RNAs (siRNAs) are effective agents [9]. Table 1 discuss the important miRNAsin various CVD along with their mechanism of action. 

This review article investigated the benefits and probable drawbacks of employing noncoding RNA vaccines as therapeutic targets in the management of CVD amidst a lot of clinical controversies along with various physiological hurdles that need to be considered while developing such formulations. A summary of key miRNAs and lncRNAs that have been linked to cardiovascular diseases are discussed. This article also covers the various delivery strategies and challenges associated with the delivery of non-coding RNAs. Last but not the least, the article presents six principles based on FDA/EUA-approved RNA-based therapy for vaccine development to be clinically relevant in the management of cardiovascular diseases. Clinicians and experimental researchers have started focusing on this particular field of therapy alongside the conventional allopathy system of medicine. Accordingly, this review article on this new field of therapy provides insight and contributes to developing future management of cardiovascular diseases. 

## 2. Therapeutics

### 2.1. Addressing Pressing Issues of CVD Using miRNA Inhibitors

As was mentioned above, miRNAs are known to control post-infarction remodelling and angiogenesis, cardiac hypertrophy and fibrosis, arrhythmias, atherosclerosis, and metabolic disorders [3,4,9,33,34]. In order to block or overexpress certain miRNAs, investigators have mostly used genetically altered mice, RNA therapies, or viral vectors to study the function of miRNAs. The major purpose of ASOs or siRNAs is to block a specific miRNA. For improved stability against RNases, both compounds have undergone chemical modification, primarily employing phosphorothioate backbones. In addition, RNAs were altered to lessen the potential for triggering an innate immune response, improve cellular absorption by improving cholesterol conjugation, and reduce off-target effects leading to improvement in the drug’s pharmacodynamics. Antisense molecules are perfect complements to their intended microRNA targets, which prevents them from coupling with their designated mRNA targets and therefore limits miRNA expression. Chemical modifications allow us to classify miRNA inhibitors, known as antimiRs, into distinct groups. The cellular uptake of certain RNAs, for example, was enhanced by increasing cholesterol conjugation, and the risk of activating an innate immune response decreased so as to improve pharmacodynamic properties. MiRNA inhibitory effect is reduced by antisense molecules that perfectly complement the target miRNA and prevent base pairing with the designated mRNA targets. MiRNA inhibitors or antimiRs, are classified according to the specific chemical modifications used to reduce off-target effects; they reduce the possibility of eliciting an innate immune response and improve cellular uptake via cholesterol conjugation so as to improve the pharmacodynamics. MiRNA inhibitory effect is reduced by antisense molecules that perfectly complement the target miRNA and prevent base pairing with the designated mRNA targets. AntimiRs are generally distinguished by the chemical modifications they cause.

### 2.2. Antisense Oligonucleotides (ASO) 

In this group, antagonists and locked nucleic acid (LNA) antimiRs are well-known participants. The oligonucleotides that make up antisense miRNAs (antagomiRs) have a structure of 2′-O-methyl, 2′-fluoro, or 2′-O-methoxyethyl accompanied by a second phosphorothioate backbone linked with the sulphur atom in place of the non-bridging oxygen atoms present in the phosphate group. The phosphorothioate backbone’s capacity to bind to plasma proteins (particularly albumin) so as to augment stability through nuclease resistance results in decreased renal clearance and better pharmacokinetic qualities. By adding a 2′-O-Me, 2′-fluoro, or 2′-methoxyethyl group, it is possible to increase the target miRNA’s ability to bind to the compound and minimise off-target effects. Cholesterol, meanwhile, increases the ability of antagomiRs to enter cells and inhibit them. The discipline of oligonucleotide chemistry has advanced significantly owing to the emergence of LNA-modified antimiRs. Linking the 2′-oxygen and 4′-carbon to form a bridge that resembles the C3′ end of a ribonucleotide is necessary for chemically locking LNAs. In a variety of in vivo studies, including those involving nonhuman primates, and clinical investigations, deoxyribonucleotide with a locked ribonucleotide sequence formed mixmers which have shown great potential [35,36]. LNA-based antimiRs are 15–16 nucleotides in length and show great specificity for the miRNA they are designed to inhibit. The 8-mer LNA-based antimiR (small LNAs) is a different LNA-based antimiR variant [37]. Small LNAs can target many miRNA families at once despite their similar functions because they bind only the miRNA seed domain. Therefore, it may be possible to increase therapeutic potential in particular disease states by simultaneously targeting each and every member of an miRNA family. One such example is a small LNA that specifically targets the miR-15 family’s seed region (miR-15a, 15b, 16-1, 16-2, 495, and 497).

In comparison with the classic LNA-based antimiR form, which only targeted a single miRNA family member, this LNA was far more efficient in derepressing downstream targets. Different short and long LNAs were all taken up by heart tissue, demonstrating that antimiR length is not necessarily a determining factor in cellular uptake. However, it appears that short LNAs are not always as efficient as the longer type of oligonucleotides [10]. In contrast to small LNAs targeted against miR-21, antagomir therapy improves cardiac fibrosis and hypertrophy while inhibiting miR-21. To enhance the performance of LNA antimiRs, two additional methods were created. Selenomethylene LNAs were distinct from the family of bicyclic RNA analogues because they had higher activity, stable metabolism, and affinities for inhibiting miR-21 in cancerous cells [38]. Another tactic is known as the “small RNA zip,” and it attempts to address related issues. Based on LNAs, short RNA zippers are designed to bridge the gap between two miRNA sequences that are only half complete due to a nucleotide gap. Using short RNA zippers, miR-17 and miR-221 were repressed in cell lines of breast cancer. However, there are currently no published reports on the potential use of either of the innovative inhibitory approaches in the treatment of CVD [39]. At last, peptide nucleic acids (a type of ASO) are an alternative to phosphate-sugar polynucleotides in which the nucleobases are connected to a flexible pseudopeptide polymer. Methyl carbonyl groups replace the phosphodiester backbone and connect the purine and pyrimidine bases to the monomer units of N-(2-aminoethyl) glycine. Peptide nucleic acids are highly resistant to DNases and proteinases and have a high binding affinity for their intended sequence. According to in vivo studies, peptide nucleic acids are effective against miRNAs, as demonstrated by the suppression of miR-155 in mouse B cells [40,41].

### 2.3. siRNAs and other Inhibitory Techniques

siRNAs are an alternative to ASOs, which are employed to silence miRNAs. These are chemically modified RNA duplexes used to enhance the nuclease stability and cellular uptake, much like antimiRs. The target miRNA is silenced when an siRNA binds to its loop region. For instance, transplanting skeletal myoblasts in mice with myocardial infarction was found to have fewer arrhythmogenic effects when miR-181a expression was reduced by employing siRNAs. MiRNA target site blockers are another targeting tactic. Due to their complementary binding, they prevent miRNAs from reaching their mRNA target region. By employing this technique, individual miRNA targets might be protected as opposed to all targets being affected simultaneously. Target site blockers demonstrated a particular reduction in miR-155’s capacity to bind to the Cebpb (CCAAT/enhancer-binding protein) gene in the hypothalamus of neonates.

Last but not least, miRNA sponges, which are generated within cells from transgenes, may be utilised to alter or reduce miRNA function. Inserted into the 3’ untranslated domain, a target miRNA can link with 4–10 complementary sites on sponge RNAs. It is feasible to select a binding site that interferes with the binding of a given miRNA to its targets by either attaching to the miRNA seed portions or basing itself on a specific mRNA target sequence. Since miRNA sponges require transgenic construct, they may be administered to tissue in living animals through viral vectors. Adenoviral eGFP (enhanced green fluorescence protein) sponges were used to downregulate miR-133 in cardiac myocytes in a mouse model depicting ventricular hypertrophy. MiRNA sponges require a large quantity to bind a large amount of endogenous miRNA in a cell [42,43,44,45]. Results from a wide range of cardiovascular studies in mice and occasionally in larger animal models, such as pigs, show that the aforementioned antimiRs effectively reduce miRNA activity in the artery wall and cardiomyoytes.

### 2.4. Antagomirs as Therapeutic Agents

Antagomirs, or anti-miRs, are a type of synthetically created oligonucleotide that aim to inhibit endogenous microRNAs (also known as miRNAs or miRs) [46]. MiR-92a antagomirs increase neovascularization following hindlimb ischemia and cardiac function recovery in mice [16,47]. Single injection (300 µg/mice; diluted in in vivo jet PEI solution) of microsphere antagomiR-92a prevented unfavourable infarct remodelling in a percutaneous pig model of reperfused AMI [18]. Furthermore, atherosclerosis and endothelial dysfunction were decreased even more by miR-92a antagonists in mice and rats [29]. In addition, AntagomiRs suppressed miR-25, which is elevated in a failing heart and shares a seed sequence with miR-92a. This treatment enhanced cardiac function, lengthened life, and slowed down the progression of heart failure in mice [27]. Later experiments with strong decoys confirmed the positive advantages of miR-25 suppression. However, a separate study found that antagomiR-25 delivered through i.p. injection of 80 mg/kg dose, spontaneously produced irregularities in cardiac function [48,49].

The notion that differences in antagomiR-25 formulation and concentration are responsible for the observed effects is still debatable. Heart function was found to improve in ischemia/reperfusion-induced injury when miR-320 is inhibited by antagomirs. Eventually, miR-212/132 antagonism reverses TAC-induced ventricular hypertrophy and failing heart in mice [50,51].

The use of LNA-based antimiRs, which suppress miRNAs, has been demonstrated and used in various disease models. Protecting against aneurysms, LNAs that target miR-29 do so by boosting matrix synthesis and preserving the arterial wall integrity [52,53]. Increased cardiac function and survival in failing hearts are additional benefits of therapeutic miR-208a inhibition with LNA-based antimiRs [54]. Suppressing the miR-34 family or inhibiting miR-34a improves myocardial infarct healing by reducing cell death and fibrosis [13,14,15]. Two types of miR-34a inhibitors, antagomiRs and LNAs, were equally efficacious in chronic myocardial infarction in mice. However, as a dose-response curve was not presented, the results should be interpreted with caution due to the fact that varying amounts of antimiR were utilised. It was also speculated that LNA-based antimiRs targeting miR-34a might increase cardiomyocyte proliferation and extend the window of opportunity for regeneration in postnatal animals. The favourable benefits of antagomiR-based miR-92a reduction were validated using LNA-based antimiRs in a pig ischemia/reperfusion model [13,15,17].

### 2.5. CVD Treatment with lncRNA Inhibition

ShRNAs, which are produced from siRNAs, modified ASOs, and gapmers, can silence lncRNAs in the same way as anti-miRs silence miRNAs. Gapmers can target nucleus rich with lncRNAs despite nuclear barriers, while small interfering RNAs (siRNAs) and ASOs preferentially target lncRNAs in the cytoplasm. To carry out something like this, ribonuclease H-dependent RNA cleavage must be introduced [6,55,56]. Since lncRNAs are mostly nuclear, antisense LNA gapmers have become the standard. These gapmers are hybrid ASOs with a deoxynucleotide monomer core long enough to initiate the RNase H cleavage critical phase. The 2′-O-modified ribonucleotides on both sides of the central block provide further defence against nuclease degradation. Gapmers are often stabilised for in vivo treatment approaches employing a phosphorothioate backbone, in the same way as other ASOs. Gapmers have been used to specifically target PCSK9 (proprotein convertase subtilisin/kexin type 9) present in the livers of nonhuman primates in in vivo studies. Nonetheless, hepatotoxicity from off-target effects halted a phase I clinical study. In contrast, no adverse events were reported over the whole year of observation of gapmers against hypoxia-inducing factor 1 [57]. Numerous studies show a potential for in vivo silencing of cardiovascular-related lncRNAs; for example, injection of gapmer, which pharmacologically suppresses the hypoxia-induced lncRNA Malat-1, decreases endothelial cell proliferation and ischemia-induced re-vascularization [58]. Inhibition of Meg3 after TAC reduced myocardial fibrosis and enhanced diastolic function, whereas gapmers against Chast dramatically reduced myocardial hypertrophy [59]. Thus, both therapy methods provide potential therapeutic options for halting heart remodelling. LincRNA-p21 and APF were both effectively used to demonstrate how lncRNAs may be inhibited by siRNAs. As was previously reported, lincRNA-p21 is a critical modulator of proliferation and cellular death in the case of atherosclerosis by suppressing p53 transcriptional activity.

Suppressing it using lentivirus-mediated siRNA release targeting lincRNA-p21 causes neointima hyperplasia in a carotid artery damage model. Damage produced by ischemia/reperfusion was mitigated in mice when the lncRNA APF, which controls the death of autophagic cells in in vitro studies, was inhibited by siRNAs [60,61]. The adequate suppression of lncRNAs in vivo requires large and frequent dosages of siRNAs and gapmers (4–20 mg/kg, several recurrent injections). Potential dose-dependent toxicities may be taken into consideration throughout preclinical and clinical testing depending on the chemistry of the inhibitor being employed. Gapmers may cause hepatotoxicity in a way that is dependent on RNase H1 in addition to the normal toxicities of RNAs [62]. 

Reducing RNase H1 levels before administering LNA-modified gapmers greatly reduced their hepatotoxicity, suggesting that off-target RNase-dependent RNA breakdown is responsible for the toxicity. Another challenge in developing lncRNA therapeutics is that, unlike miRNAs, lncRNAs are not necessarily conserved across species. 

Therefore, it is crucial to carefully organise and execute animal experiments that focus on the action mechanism of a specific lncRNA, as well as studies on toxicity, before adopting the findings in the clinic. One option is to employ the human-specific gene instead of the endogenously expressed sequence in animal models of toxicology. However, this has the downside of limiting research into the harmful effects of a gapmer to only those that do not depend on hybridization. Humanized models or organoid cultures may be useful for elucidating the role of lncRNAs in humans. This is an important step since lncRNAs’ effects on chromatin structures or epigenetic regulatory processes may be nuanced, and the development of lncRNA therapeutics may be hampered if the underlying mechanism is not understood. Most mRNAs and miRNAs are found in the cytoplasm, whereas lncRNAs are primarily expressed in the nucleus, where they may be a part of inaccessible complex structures. Small compounds intended to selectively interfere with conserved RNA structures, such as those that disrupt RNA protein complexes, may be advantageous. Table 2 summarises some crucial lncRNAs in CVD.

## 3. Pharmaceutics of RNA-Based Vaccine Delivery

### 3.1. Hurdles in the Systemic Delivery of siRNA

Whatever may be the biochemical and molecular mechanism, all RNA payloads must avoid off-target organ clearance, enter the target tissue, interact with the appropriate cell type in a dynamic microenvironment, be taken up via endocytosis, and then escape the endosome without triggering a detrimental immune response. Unlike ASOs, siRNAs, and other short RNA therapies, mRNA and DNA-mediated therapies need a vehicle for cellular entry. Numerous polymer- and LNP-based RNA delivery technologies have been developed by researchers. We have discussed the problems that come with using siRNA.

### 3.2. Stability in the Circulatory System

When injecting siRNA into a vein, stability in the circulatory system should be the primary concern. Naked siRNA is easily broken down by several endogenous enzymes and can become aggregated together by serum proteins in the circulation, thus it is important that the siRNA delivery mechanism is "cohesive" enough to prevent this. Surface qualities that facilitate engagement with the intended cellular targets must be presented by the delivery method while simultaneously limiting nonspecific opsonization, phagocytosis, and eventually immune triggering. Since naked siRNA gets cleared from the bloodstream within 5 min of intravenous delivery, its circulation must be maintained for it to be effective [73,74,75,76,77].

### 3.3. Vascular Endothelium as the Semiselective Barrier

The endothelium controls the diffusion of substances into tissues by serving as a selective barrier between the arterial lumen and surrounding tissue. Basically, there are three types of normal capillary endothelium: continuous endothelium, fenestrated capillaries, and discontinuous capillaries [78]. To achieve a high capacity for crossing the barrier posed by endothelium, the siRNA delivery size should be less than 150 nm, since this is the size limit imposed by the normal endothelial structure. Retention and enhanced permeability are the two factors on which the efficacy of a drug largely depends. Modifying the localised shape of blood arteries can also affect the efficacy of drug delivery. Nanoparticles (NPs) with a size of 500 nm or less, and often less than 150 nm, showed dramatically augmented penetration and retention properties in "leaky" tumour blood arteries [79].

### 3.4. Extracellular Matrix Diffusion 

The extracellular matrix (ECM) is a complex network of polysaccharides and fibrous protein gels that surround and support cells. The siRNA-loaded vehicle must first cross the vascular endothelium before entering the ECM. Larger NPs have trouble penetrating tumour tissue due to the tight nature of the ECM. Since nanocarriers are so tiny, they are better able to transport siRNA into tissues that have low permeability. Furthermore, NPs’ charges may potentially have an impact on particle diffusion. It has been shown that the diffusion of neutral particles is quicker than the charged ones [80].

### 3.5. Cytoplasmic Delivery

For siRNA to be effective, the delivery mechanism must first cross the cell membrane, then enter the cytoplasm, and finally discharge its cargo. Due to its negative charge, however, siRNA has a hard time crossing the negatively charged cell membrane. Endocytosis mechanisms such clathrin-mediated endocytosis (CME), macropinocytosis, and caveolae-/lipid raft-mediated endocytosis are typically used by nanocarriers to deliver siRNAs cargo into cells [81,82]. After macropinocytosis, siRNA is typically delivered to the endosome where it is degraded enzymatically. Once in place, the siRNA transporter quickly fuses with lysosomal vesicles. Consequently, successful siRNA dispersion also needs endosome egress.

Compared with macropinocytosis and CME, caveolae-/lipid raft-mediated endocytosis is a less well-understood process [83,84]. Direct cytosolic administration of the therapeutic agent, via the lipid raft mode, has been proven to be possible and may provide a novel method for improved cytosolic siRNA delivery [85,86,87]. 

A variety of non-viral siRNA delivery vehicles have been fabricated to avoid systemic delivery problems. These include polymers or lipid polymer hybrid NPs, lipid-based NPs, hydrogels, microbubbles, inorganic NPs such as gold, quantum dots, silica, carbon nanotubes, iron oxides, exosomes, and oligonucleotide NPs [88,89,90,91,92,93,94,95,96,97,98,99,100,101,102,103,104]. When compared with inorganic carriers and viral vectors, lipid-based NPs stand out as having superior biocompatibility and biodegradable qualities.

## 4. Delivery Strategies to Improve the Targeting and Therapeutic Efficacy of Non-Coding RNA-Based Vaccines

There are still obstacles to overcome despite the extensive study of oligonucleotide-based therapeutics targeting shRNA (short hairpin RNA) and lncRNAs in the cardiovascular domain. There are two major aims. The goal is to lessen the amount of drug needed to block noncoding RNAs in cardiovascular tissues and reduce hybridization-independent toxicity. The toxicity and sequence-specific adverse effects of using antisense therapy can be mitigated through targeted delivery to specific cell types. Dose-dependent toxicities and off-target effects can be mitigated by using more efficient delivery techniques which increase cellular uptake or cell-type selectivity. A ubiquitously expressed miRNA may perform beneficial and detrimental effects in different cell types. Therapeutic overexpression or inhibition of an miRNA requires targeting the proper cell type. MiRNA inhibitors, such as those belonging to miR-15 or miR-34 families, increase cardiomyocyte proliferation and heart regeneration [11,13]. Such a strong miRNA inhibitor will likely enhance division in other cell types, thereby promoting tumour growth, while stimulating cardiomyocyte proliferation may have therapeutic effects and allow for prolonged heart regeneration. MiR-34 and miR-15 limit tumour development, and miR-34 overexpression are looked into for possible cancer treatment [105,106,107]. Below we have discussed some drug delivery options for noncoding RNAs. 

### 4.1. Lipid and Lipid-Based Nanoparticle Vaccines

**Figure 1:**
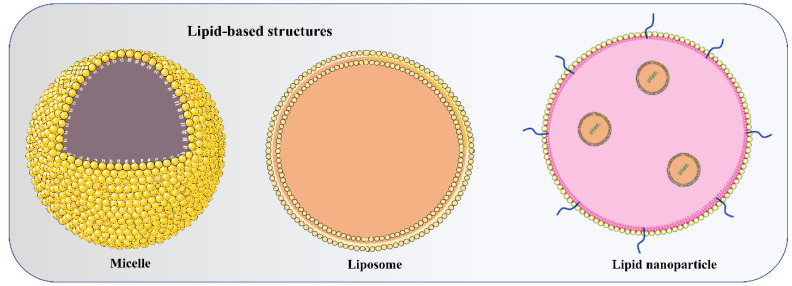
Depicts the basic structure of micelle (monolayer), liposome (bilayer), and lipid nanoparticle (LNP) (multiple lipid layers with microdomain) used to deliver non-coding RNAs to target cells.

**Figure 2:**
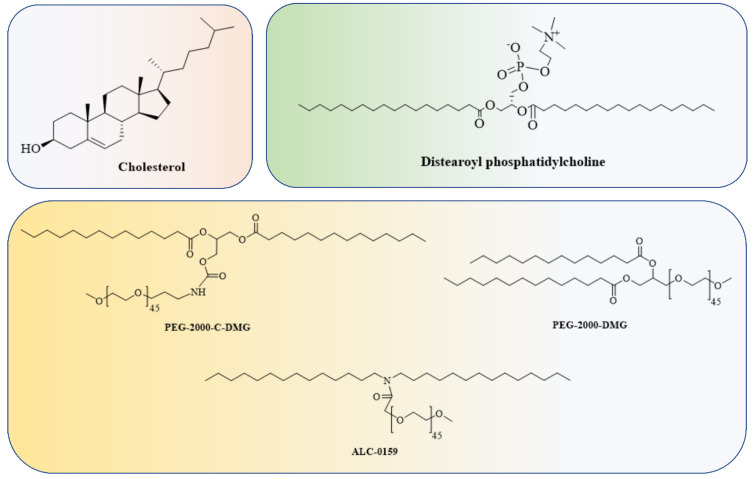
Cholesterol, a helper lipid, a PEG-lipid, and the RNA payload are common components of LNPs.

**Figure 3:**
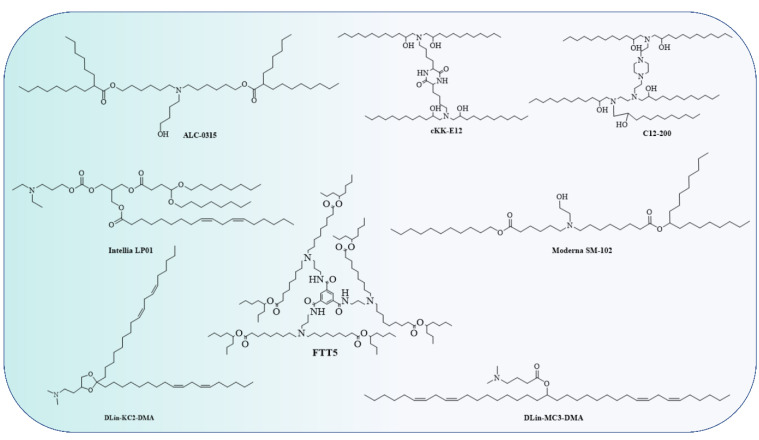
Though the lipids have different chemical structures and chain lengths, the presence of a tertiary amine at acidic pH takes a proton and turns it into a cation, i.e., a cationic lipid. This cationic lipid binds to the anionic part of RNA, forming a stable LNP.

The Food and Drug Administration (FDA) has approved the use of LNPs (lipid nanoparticles) for the transport of siRNA to the liver [108] and mRNA vaccines [109,110]. Micelles, liposomes, and LNPs (Figure 1) are all different forms of lipids differentiated by the overall size of the hydrophilic head group and hydrophobic tail or tails [111]. Each of the four major components (cationic or ionizable lipid, helper lipid, cholesterol, and poly(ethylene glycol) (PEG)-lipid) (Figure 2) constitute FDA-approved LNPs. Studies using lipid-based delivery systems complexed with nucleic acid [112,113] indicate that lipid structure modifies LNPs’ interactions with cells [114]. Hundreds to thousands of chemically distinct lipid delivery systems [115,116] have been developed as a result of the fact that lipid structure influences the delivery of nucleic acids and that such systems can be easily produced using Michael addition-based, epoxide-based, and alcohol-based chemical reactions. Many of these studies aimed to enhance the delivery of siRNA to hepatocytes or the liver cells of mice [117]. In conjunction with a more rational approach to lipid design [114], these investigations reduced the dosage required for robust in vivo gene silencing in the hepatocytes of mice from around 1.0 mg/kg [118] to 0.002 mg/kg. DLin-KC2-DMA is an ionizable lipid [114]; cKK-E12 is a peptide-like lipid compound [119]; DLin-MC3-DMA [120] was used in patisiran to treat hATTR [108]; and C12-200 was synthesised using an epoxide–amine reaction [117], LNPs have been used to transport messenger RNA (mRNA) to the livers of mice, non-human primates, and humans. Some LNPs made use of lipids that have been optimised for siRNA transport. For instance, mRNA was transported to the liver in mice using LNPs in conjugation withcKK-E12 [121,122], C12-200 [123], and DLin-MC3-DMA [124]. mRNA has also been transported to the mice hepatocytes by more recently reported lipids such as LP01 [125] (Intellia Therapeutics), Lipid H [126] (Moderna), and FTT5 [127] (Ohio State and Beam Therapeutics) (Figure 3).

Two LNPs made using an undisclosed cationic or ionizable lipid, PEG-lipid, cholesterol, and 1,2-distearoyl-sn-glycero-3-phosphocholine (DSPC) were recently used to transport mRNA expressing a base-editing Cas9 and sgRNA targeting PCSK9 to the liver in non-human primates [128,129]. After a single injection of LNP, the PCSK9 gene was silenced permanently. Long-term PCSK9 inhibition with antibodies [130] or siRNA [131] has been shown to improve CVD in humans. Beam Therapeutics independently reported long-lasting effects in the livers of non-human primates after LNP-mediated base editor delivery [132].

Complementing prior preclinical studies, Intellia has also disclosed data in patients dosed with NTLA-2001 to inactivate the TTR gene. There is evidence that TTR inactivation is effective in people, as therapy with siRNA or ASOs targeting TTR decreased the development of hATTR amyloidosis with polyneuropathy [133,134]. Alnylam, Moderna, and Pfizer/BioNTech/Acuitas LNPs each include four extra components in addition to their respective RNA payloads: cholesterol; the PEG-lipids PEG-2000-C-DMG (Alnylam), PEG-2000-DMG (Moderna), or ALC-0159 (Pfizer/BioNTech/Acuitas); and DSPC. While the majority of preclinical research has focused on how the structure of cationic or ionizable lipids influences transport, the other three components may also have an effect [135,136]. An LNP that was initially designed to deliver small interfering RNA (siRNA) to endothelial cells in the lungs and blood vessels of mice [137] and non-human primates [138] has been retargeted to deliver siRNA [139], small guide RNA (sgRNA), or messenger RNA (mRNA) [140] to endothelial cells in the bone marrow, liver, and spleen following intravenous administration and lung epithelial cells after nebulization. Other examples include how altering the PEG-lipid structure or its molar fraction influences LNP pharmacokinetics and hepatic siRNA delivery in mice [141,142,143,144]. The lipid “anchors” the PEG-lipid inside the LNP, whilst the hydrophilic PEG interacts with blood plasma water to produce an aqueous barrier [145].

In a similar way, oxidised cholesterols [121], esterified cholesterols [140], and cholesterol analogues such as phytosterols [113] have all been reported to enhance LNP dispersion in cell cultures and in mice, despite the fact that the majority of LNPs are formed from unmodified cholesterols. The addition of modified cholesterol to LNPs may affect their structure [146], but the mechanism by which cholesterol mediates delivery enhancements remains unknown. It has been found that substituting a different lipid for DSPC improves LNP transport to the spleen and lungs [122,147]. Similarly, LNPs were targeted to the lung and spleen by selective organ targeting [148] by adding another lipid to the LNP and therefore changing it from a four-component to a five-component system. Beam Therapeutics [132] and Intellia [149] have published data demonstrating that LNPs can be manufactured to transport mRNA to haematopoietic stem and progenitor cells in mice, a discovery that speaks well for the development of in vivo haematopoietic stem-cell-targeting therapeutics.

MiR-153-3p is protective against ischemia/reperfusion injury, although its role in myocardial infarction (MI) is uncertain. Liposome nanoparticles and HA-cationic liposomes (CLPs) for miR-153-3p transport and mechanistic activities of miR-153-3p were modified by nHA-CLPs in MI-induced cardiomyocyte injury. miR-153-3p protected cardiomyocytes against apoptosis and damage generated by MI. It was observed that miR-153-3p binds to the 3’ untranslated region of KLF5 (Kruppel-like factor 5) and suppresses its production. The inhibition of NF-κB by miR-153-3p reduced inflammation [150].

### 4.2. Polymer-Based Nanoparticle Vaccines

**Figure 4:**
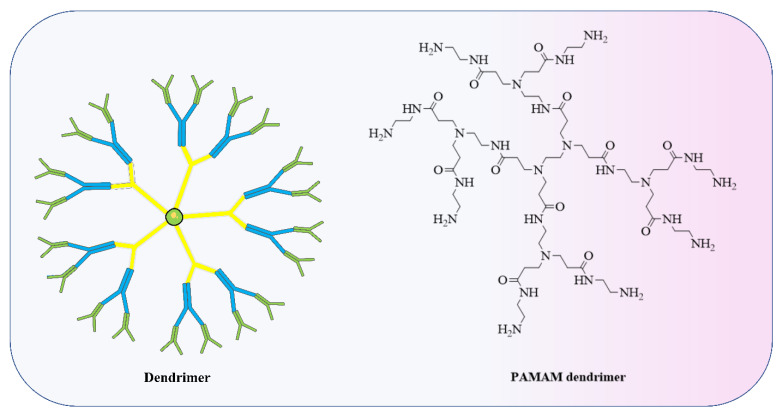
Dendrimers are specific polymeric structures made up of a set number of molecules arranged around a central PAMAM, poly(amidoamine) used to form LNP.

**Figure 5:**
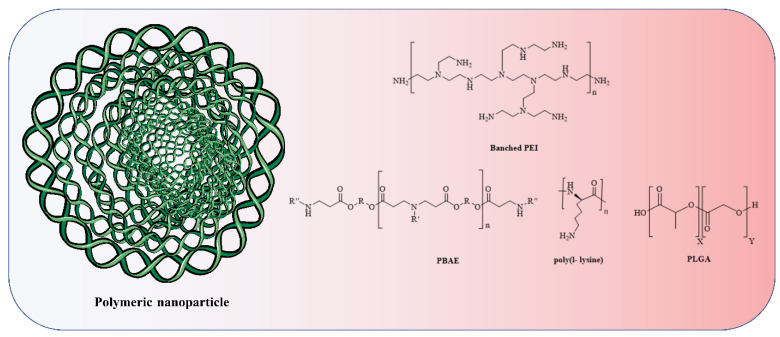
The anionic phosphodiester part of RNA is complexed by cationic amine groups (due to attachment of a proton) in polymeric nanoparticles and polymers based on poly(ethylenimine) (PEI), poly(l-lysine) (PLL), and poly(beta-amino ester) (PBAE). Polymers derived from poly(lactic-co-glycolic acid) (PLGA) are commonly modified to incorporate discrete cationic groups.

Furthermore, polymers and polymeric nanoparticles are used in several non-viral RNA delivery techniques, especially dendrimers (Figure 4) [151]. In order to modify how polymers transport RNA into cells, chemists can adjust properties including charge, degradation rate, and molecular weight [152,153]. Poly(lactic-co-glycolic acid) is a conventional polymer (PLGA). PLGA drug delivery systems have been approved by the FDA for the delivery of small-molecule therapeutics but not for nucleic acids [154]. PLGA (Figure 5) does not possess a positive charge which is important to form a complex with the anionic RNA phosphodiester backbone at neutral pH. Therefore, researchers have incorporated cationic groups into the PLGA moiety, such as chitosan, to facilitate the transport of siRNA in mice [155]. Preclinical investigations of several CVD have shown promise for the use of siRNA-based nanoparticle platforms, such as liposome-RNA complexes and polymer-based RNA complexes [156]. Possible strategies for selectively targeting active endothelium include liposomal nanoparticles (cationic amphiphiles) loaded with antibodies against VCAM-1 or E-selectin for siRNA delivery to activated primary ECs in vitro [157]. Conjugation of siRNA has seen extensive use of polymeric forms, knocking down the advanced glycation end products (RAGE) receptors through direct injection into infarcted regions in myocardial infarction (rat model) successfully. A conjugation of deoxycholic-acid-modified polyethylenimine and siRNA was used against the RAGE mRNA efficiently silencing its expression, eventually resulting in decreased levels of inflammatory cytokines, in vivo apoptosis, and ventricular remodelling [158]. A silica nanoparticle method was formulated to deliver miR-24 in AMI (acute myocardial infarction). Dithiobis(succinimidyl propionate) (DSP) was coupled with polyethylenimine (PEI), followed by functionalized silica nanoparticles (F-silica), which noncovalently changed DSP–PEI on the surface of the nanoparticles; this was achieved using the reverse microemulsion technique. Finally, an F-silica-miR-24 gene carrier complex was produced. Nanoparticles had substantial cellular uptake and retained miR-24’s function. F-silica-miR-24 was shown to be a successful miR-24 replacement therapy in primary cardiomyocytes from rat models of AMI, with favourable biocompatibility. These results suggest that regulating miRNA expression during stress-induced apoptosis could be a useful therapeutic strategy for treating CVD. F-silica-miR-24 vectors are easily uptaken, biocompatible, and exhibit 78% gene transfer efficiency. It did this by evading degradation in endolysosomes and releasing miRNA molecules, which then disrupted the target gene (Bim) in cultured cardiomyocytes. Reduced ventricular remodelling with long-term improvement in cardiac function were observed in mice with the F-silica-miR-24 delivery method in early cases of acute myocardial infarction due to Bim inhibition [159]. Nanoparticles having a stable shelf life and a size appropriate for the physiological delivery of miR therapies in vivo are required. The miR nanoparticles containing the cardioprotective miR-199a-3p were synthesized with a DSPE-PEG shell and a CPP conjugation. The fluorescent core of the miNP was made of poly(9,9-dioctylfluorene-co-benzothiadiazole), which allows particle identification and tracking. MiNPs typically measured approximately 110 nm in size, with little to no fluctuation, making them comparable in size to exosomes that are produced naturally (30–120 nm). The miNPs were stable in terms of size, surface charge, and dispersion even after being subjected to many freeze–thaw cycles. Since miNPs exhibited little cellular toxicity, the delivery to cardiomyocytes at higher miRs concentrations can be achieved without causing damage [160]. Transfection efficiency is low when using nonviral vectors in the case of nondividing cells such as cardiomyocytes. The TAT CPP was integrated into the miNP shell to resolve these uptake problems. The transactivation motif of HIV-1 (TAT) is a cationic moiety that facilitates HIV-1 absorption into cells. Uptake of miNP by human embryonic stem cell (hESC)-derived CMs and hESC-derived endothelial cells (ECs) was enhanced by the addition of TAT CPP (ECs). These results demonstrate the feasibility of nonviral miR delivery to cardiac tissues, which had been an earlier challenge but is now seen as promising for in vivo translation of the treatment [161]. Mimicking cardiac macrophages after MI, miRNA-21 given via nanoparticles reduces inflammation. Upregulation of miRNA-21 by peritoneal macrophages after apoptosis reduces inflammation. Thus, miRNA-21 was formulated as nanoparticles (NPs) due to the formation of a complex of hyaluronan-sulphate (HAS) and nucleic acid via calcium ion bridges. Laser capture microdissection (LCM) was used to monitor the LV posterior wall. LCM allowed researchers to study cardiac macrophages in their native milieu without processing of cells or in vitro culture affecting their activation. After coronary ligation in mice, cardiac macrophages expressed miRNA21. MiRNA-21 has inconsistent effects on macrophage polarisation according to studies. MiRNA-21 is upregulated within macrophages after engulfing cells apoptotic in nature, enhancing inflammation resolution, while pri-miR-21 controls early inflammation. This modulation increases angiogenesis, reduces apoptotic cells, and slows left ventricular remodelling following MI. Current modulation did not enhance systolic function and will likely need more improvement to do so [162]. Whether or not Au nanoparticles might be functionalized with a ligand capable of attaching firmly and precisely to a biomarker of endothelial cells (disturbed flow, or d-flow) was investigated. The presence of VCAM1 on inflamed endothelial cells prompted the functionalization of Au nanoparticles with a VCAM1-binding peptide (VHSPNKKGGSKGC) to minimise non-specific accumulation and negative consequences due to the presence of VCAM1. The highly flow-sensitive pro-atherogenic miR-712 is increased in endothelial cells in response to d-flow, as established by in vitro and in vivo studies. In this study, Au nanospheres of 5 nm size were used for targeted delivery of anti-miR-712 to inhibit miR-712 expression and activity. Using a complementary DNA carrier, anti-miR-712 was hybridised to an Au nanosphere. Derivatization of Au nanospheres with mPEG-SH and NH2-PEG-SH at varying ratios reduces the protein corona, subsequently functionalizing the nanospheres with carrier DNA that has a 3’-end thiol group and 10 adenine (A) nucleotides. Following this, anti-miR-712 is hybridised to the Au nanosphere through the carrier DNA. To prevent its release into the body’s extracellular fluid inadvertently, anti-miR712 only weakly attaches to carrier DNA. Because of improved interaction with the carrier DNA, anti-miR-712 is quickly released inside cells after being introduced. For targeted delivery of anti-miR-712 to active endothelial cells, a VCAM1-binding peptide might be incorporated into Au nanospheres [163].

Optimising multifunctional nanoparticles is difficult because they need a high loading efficiency, a hidden cationic domain to enable lysosomal escape, and dense, stable, targeting moieties. Vascular cell adhesion molecule 1 (VCAM1) was targeted by loading anti-miR-712 (1400 molecules, >95% loading efficiency) onto cationic lipoparticles (CCLs) coated with 5 mol% of peptide (VHPK). In vitro and in vivo studies confirmed disease-specific accumulation of anti-miR-712 in endothelial cells from inflamed mice aortas. With the help of VHPK-CCL-anti-miR-712, the endothelium’s metalloproteinase activity was suppressed by reducing the miR-712 expression that was increased by d-flow and restoring the expression of its target genes, TIMP3 and RECK. VHPK-CCLs transported anti-miR-712 to inflamed endothelium in pro-atherogenic d-flow areas. This may also be used in case of mimics, miRNA inhibitors, plasmids, siRNAs, and pharmaceuticals. VCAM1-targeted cationic nanoparticles containing anti-miRs can be used for tailored antiatherogenic treatment with little off-target effects [164]. Functional miRNA mimics were delivered to macrophages in vivo by chitosan nanoparticles (chNPs). chNPs, a cross-linked chitosan polysaccharide polymer, have been shown in a number of in vivo and in vitro investigations to potentially act as a shield and transmitter of exogenous miR-33 to naive macrophages, hence influencing ABCA1 expression. Macrophages treated with chNPs expressing miR-33 exhibited reduced cholesterol efflux to apoA1 and reduced RCT. Delivery of the efflux-promoting miRNAs miR-206 and miR-223 into cells via nanoparticles led to elevated expression of ABCA1 and the RCT pathway. With the results of this study in hand, it is possible that miRNAs might be employed in vivo to selectively target atherosclerotic lesions by being transported to macrophages by chNPs and regulating ABCA1 expression and cholesterol efflux [165].

### 4.3. Device-Based Methods for RNA Vaccines 

As a preliminary attempt to boost concentration in the heart or vasculature, local RNA treatment delivery devices have been tested. A pig ischemia/reperfusion model was utilised to test the efficacy of LNA-92a at doses of 0.03 mg/kg b.w. intravenously, intracoronally (antegrade), and retrogradely. While miR-92a expression was reduced by all three treatment routes, antegrade and retrograde infusions were more effective at enhancing heart function and decreasing apoptosis as well as infarct size than intravenous administration [17]. The LNA concentration in this study was low (0.03 mg/kg b.w.), especially when compared with previous studies utilising LNAs (0.5–25 mg/kg b.w.), and further increasing the dose to 0.15 mg/kg (intracoronary injection) did not further boost therapeutic effectiveness [53,54,166]. Injecting antimiRs by catheter into the heart successfully reduced the systemic inhibitory effects of miR-92a, but this was not the case for other miRs. Intramuscular injection is an alternative delivery strategy for RNA therapy since it has been shown to be successful in cell therapy. Another option is to employ drug-eluting stents or balloons to deliver RNA therapies to the arterial wall. It has been shown that anti-21-coated stents can locally decrease miR-21 expression just as effectively as systemic miR-21 inhibition, with fewer adverse effects [167].

### 4.4. RNA Viral Vector-Mediated Vaccine Delivery

Moreover, tissue-specific accumulation of miRNAs or lncRNAs can be accomplished by the use of viral vectors for cell-type-specific delivery [168]. For example, AAV serotype 9 has been utilised to overexpress miR-199a and miR-590 in cardiomyocytes for an extended period of time [169]. One further use of this idea is the restoration of miR-1 levels to prevent ventricular hypertrophy [24]. Cardiomyocytes are the only cell type that AAV serotype 9 viruses target, hence these viruses have been widely utilised in small animals to overexpress miRNAs, shRNAs, and mRNAs [24,43,169,170]. While there is hope for AAV-mediated gene therapy, its lacklustre performance in human clinical research (CUPID trial (Calcium Upregulation by Percutaneous Administration of Gene Therapy in Cardiac Disease)) implies there is substantial room for improvement in vector efficiency. If vasculature-specific tools are considered, the methods are less sophisticated [171]. Though vectors targeting endothelial cells do exist, they have so far only been shown to have an effect on the liver and not the endothelium lining the heart [172]. A novel strategy for introducing viruses to tissues has just been reported in the scientific literature. A technique called split-intein-mediated protein trans-splicing was used to attach large polypeptides to the AAV particle surface by covalent bonding. Gene transfer to specific cell types was achieved by the use of scFvs (single-chain variable fragments) or DARPins (designed ankyrin repeat proteins) that have a high affinity to cell surface receptors expressed exclusively on the cell membrane of target cells. Furthermore, compared with AAVs expressing the same targeting ligand but not genetically connected, protein trans-splicing-based AAVs transmit far less of their genes into target receptor-negative cells [173].

### 4.5. Vaccine Based on Tissue Enrichment via RNA Therapeutics Modification

Chemically modifying the ASO is another option for improving tissue-specific absorption of RNA therapies or generating locally restricted activity. Improved wound healing was observed in diabetic mice when miR 92a was inhibited by light-induced RNA interference (RNAi) [174]. Light-inducible antimiR-92a’s activation and delivery has been demonstrated to be equally effective as conventional, non-activatable antimiRs at decreasing miR-92a expression in mouse skin. The levels of miR-92a in other organs were not significantly altered by local activatable antimiRs, in contrast to conventional antimiRs. Light induction of RNA treatments needs more study before it can be employed on well-perfused visceral organs such as the heart. It is conceivable to enrich a specific antimiR or miRNA mimic in a cell-type-specific manner by linking miRNAs to aptamers. Tumour models and endothelial cells have both benefited from aptamer-mediated miRNA delivery. One study established a connection between miR-126 and an aptamer that binds the widely expressed transferrin receptor [175]. Currently, there is insufficient information to conclude that this is even possible in an in vivo cardiovascular system. Transferrin receptor miR-126 chimeras, or the delivery mechanism enhanced with additional endothelial-specific aptamers, are promising therapeutic options for the treatment of vascular disorders. Using aptamers to target a specific cell population and microparticles to enhance cellular uptake of miRNA mimics proved effective in treating atherosclerosis. Specific miR-181b and miR-146a delivery to activated endothelial cells was achieved by loading nanoporous microparticles constructed of porous silicon multistage vectors [19].

### 4.6. miRNA Encapsulation

Improving cellular uptake of RNA treatments by encapsulating the pharmaceuticals might be an option. Many different approaches of molecular targeting have been studied experimentally so far. Upon intracoronary administration in pigs, antagomir-92a made of 9 μm poly-d,l-lactide-co-glycolide microspheres selectively target the vasculature and improve functions of the heart [18]. Overexpression of miR-126-5p was shown to be possible using miRNA mimics delivered in a nanoparticle-based system. MiR-126 upregulation inhibited atherosclerosis and rescued endothelial cell proliferation [176]. Atherosclerosis development was inhibited through a cell-specific route when miR-181b mimics conjugated with lipofectamine were injected into the vascular endothelium [177]. In the aforementioned investigation, overexpression of miR-146a and miR-181b as a treatment for atherosclerosis was tested by delivering the RNAs to the inflammatory endothelium that surrounds atherosclerotic plaque using a multistage vector that targets E-selectin. Polyethylene glycol-polyethyleneimine nanoparticles contained multistage vector microparticles targeting E-selectin loaded with Cy5-conjugated miR-146a and miR-181b [19]. An intracardiac vaccine based on a synthetic lipid delivery system and loaded with hsa-miR-199a-3p and hsamiR-590-3p mimics was shown to enhance ventricular function just after an episode of AMI. Such a synthetic miRNA lipid drug delivery vaccine system is sufficient enough for the restoration of cardiac functions [178]. However, there are currently few established protocols for improving noncoding RNA therapeutics in cardiac tissue, especially ASOs, despite the aforementioned promising instances. In contrast, hepatic tissue may be the focus of LNP (lipid nanoparticle) administration methods using ionizable amino lipids, which have recently emerged as the principal siRNA delivery approach with many products now in clinical trials. siRNA-loaded LNPs were able to cause silencing of genes in the hepatocytes when its vaccine was injected intravenously at low doses of 0.005 mg/kg b.w. in animals [120]. The liposomal nanoparticle vaccine formulation known as SMARTICLES, with a size of roughly 120 nm, has been used in phase I clinical studies to deliver the tumour-suppressing miR-34. As for efficacy, biodistribution, and safety, all were maximised compared with other delivery strategies [179]. The efficacy of the vaccine formulated by conjugating ASOs with GN3 (N-acetyl galactosamine), a high-affinity ligand for the hepatocyte-specific ASGPR (asialoglycoprotein receptor), was tested in the liver of mice and was found to increase by 6–10 fold [180].

## 5. Challenges Associated with RNA Vaccine Therapy

Although miRNA inhibition has shown therapeutic advantages in mice and large animals, targeting cardiac tissue is more difficult than kidney, liver, or other organs of interest [181]. The anti-imiRs vaccination can be given intravenously, intramuscularly, or subcutaneously for long-term inhibition. Studies in mice or rats using LNA-based antimiR typically used doses between ≈ 0.5 and 25 mg/kg. To suppress miRNAs in cardiomyocytes or the vasculature, however, significantly greater doses of AntagomiR (8–80 mg/kg body weight) were required. Both chemical composition and the sequence of targets might affect the appropriate doses and dosing strategy (the number of injections). Studies in mice showed that very low doses of LNA-92a (0.5 mg/kg) or antagomiR-92a (8 mg/kg) were sufficient to effectively suppress miR-92a in the cardiomyocytes [17]. Targeting miRNAs such as miR-34a or miR-29 required a significantly higher dose (5 mg/kg LNA-based antimiRs, 20 mg/kg antagomiRs), despite the fact that the tissues they acted on were the same [13,53]. In contrast, vaccines formulated with siRNA delivery have shown efficacy at concentrations as low as 0.01 mg/kg bodyweight, which is essential to inhibit the hepatic genetic expression [120], whilst in the first clinical study, 3–7 mg/kg of body weight was applied to an antimiR that targets the miR-122 that is highly expressed in the liver (5 weekly injections) [120,182]. The appropriate dose of a given miRNA inhibitor appears to be affected by a number of factors. Both the miRNA and the inhibitor sequence play crucial roles. Furthermore, biodistribution and bioavailability, cellular uptake process, targeted miRNA, and associated genes are also important. By developing novel chemical changes, bioavailability was increased by decreasing clearance from the circulation. This means that antimiR activity is no longer restricted to highly accumulated tissues such as the liver and kidney, but may be found in a wider variety of organs. It is unclear, however, whether or not assessing ASO uptake alone is connected to their biological action [183]. The endocytotic mechanisms that are responsible for the ASOs uptake can be classified as either productive or nonproductive. Although uptake via productive routes results in ASO binding to its target, uptake via nonproductive pathways may cause accumulation of the ASO in late endosomes or lysosomes [184]. Even if the mechanisms behind the route towards production as opposed to nonproduction uptake are not entirely understood, TSG101, a component of the ESCRT-I (endosomal sorting complexes required for transport I), is a critical modulator of nonproductive uptake of anti-miR-21 [185]. Productive uptake was enhanced in both in vitro and in vivo trials when TSG101 was inhibited. Evidence suggests that surface protein expression and binding capacities regulate the uptake process [184]. Furthermore, the effective dose may change depending on how much the corresponding target genes and miRNAs are expressed. In the past, evaluating knockdown effectiveness frequently required removing the RNA from the entire tissue, which could produce inconsistent results. Since the presence of the antimiR in the tissue may directly modify the PCR, this method of evaluating miRNAs may overestimate the inhibitory effects. 

Therefore, Northern blotting should be employed to validate the level of inhibition, with a focus on evaluating target derepression. Additionally, assessing the actual biological significance of miR inhibition may be complicated by cellular heterogeneity. The heart is composed of many different cell types, including cardiomyocytes, endothelial cells, immune cells and fibroblasts, each of which can express miRNAs and their targets uniquely. This can make it challenging to discern effects in whole heart tissue. It may be required to examine the effects of antimiR therapy on a subset of cells. The expression levels of miRNAs and their targets can undergo drastic alterations in pathological circumstances.

The potential for harmful effects from ASOs and antimiRs increases with the dose. Hybridization-dependent toxicity and hybridization-independent toxicity are the two main types of toxicity seen after oligonucleotide therapy. The sequence of the oligonucleotide is responsible for the toxicity caused by hybridization, which can be caused either by direct modulation of on-target effects or nonspecific binding with other nucleotide sequences with comparable characteristics (off-target effects). Preclinical and clinical experiments take into account the target’s mechanism of action and expression profile/level, and toxicity caused due to it can be evaluated and possibly mitigated. Off-target effects in miRNAs could be caused by the seed sequence being too similar to the target RNA or by random interactions with other molecules.

Although studies have been conducted on antimiRs that target the 3′ end of the miRNA, the results have been widely contested, making it unclear which approach is most successful [37]. Furthermore, targeting of an entire miRNA family, such as in the case of miR-21 where conflicting outcomes were found, may be advantageous but may not be appropriate for other miRNAs [186]. Hybridization to double-stranded DNA sections might further produce off-target effects, possibly generating site-specific mutagenesis through the triplex formation. Standard assays, including AMES and micronucleus testing, found no evidence for genotoxicity in a panel of oligonucleotides, albeit this outcome is theoretically plausible [187]. Newly modified oligonucleotides should still be subjected to genotoxicity testing. Because of the oligonucleotide’s base pair structure and any alterations made to it, it can be hazardous even when not hybridised. Conventionally, RNA oligonucleotides stimulate the immune mechanism by way of TLR (toll-like receptor) signalling [188]. Phosphorothioate-modified oligonucleotides activate the complementary system in non-human primates, particularly at high plasma concentrations [189,190,191]. The peak plasma levels that cause these adverse effects can be minimised with gradual infusion as opposed to bolus administration. Human serum did not show any evidence of complement activation in response to oligonucleotide therapy, which is interesting. Platelet activation, aggregation, and thrombus formation have all been demonstrated to occur in vitro and in vivo when phosphorothioate-modified oligonucleotides bind to platelets [192]. Recently, it was shown that blocking ASO binding to platelet proteins by LNA insertion significantly attenuated platelet activation [193]. However, it is crucial to incorporate adequate immunologic testing into both the preclinical and clinical phases in the development of the pharmaceutical. Raised levels of the transaminases alanine aminotransferase (ALT) and aspartate aminotransferase (AST) present in liver have been associated with the use of several LNA-modified oligonucleotides which is an indicator of possible hepatotoxicity [194]. Hepatotoxicity can be attributed to a specific sequence rather than a conventional one, as several LNA-based oligonucleotides exhibited non-toxic effects in the liver (in vivo). Trinucleotide motifs, which increase p53 and NRF2 (nuclear factor [erythroid]-derived 2]-like 2) pathway activation in vivo, are intriguing because they may be predictive of potential hepatotoxicity [195].

## 6. Six Qualities of Vaccine Delivery Systems to Be Clinically Relevant 

**Figure 6:**
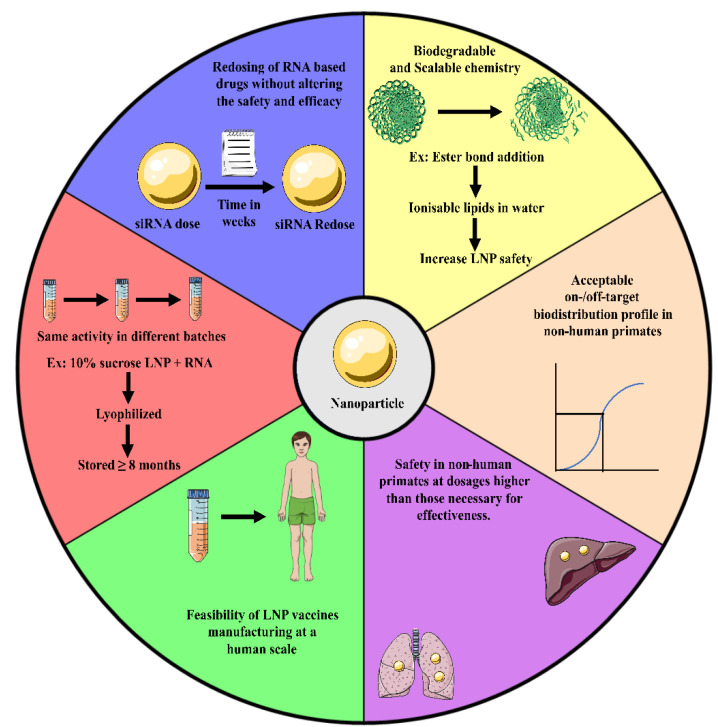
Any RNA-based vaccine delivery during its development must possess the 6 mentioned ideal characteristics (approved by FDA/EUA) so as to exert clinical relevancy and reach the pharmaceutical market.

There has been a constant effort to develop nanoparticle-based RNA delivery using reported vehicles. A detailed pharmacokinetic and clearance clinical data have been reported regarding patisiran [196,197]. The approved animal models, toxicity readouts, and time points for patisiran were detailed in a report from the FDA Center for Drug Evaluation and Research [198]. These data provide a thorough roadmap for characterising nanoparticle-based RNA delivery of appropriate clinical outcome. In a broader sense, the 3 RNA drug delivery systems that have received FDA approval [108,199] or an EUA grant [200] thus far seem to have six qualities (Figure 6) that, when taken together, might be considered hallmarks of authorised delivery vehicles. To begin with, biodegradable, scalable chemistry is typically used in the fabrication of therapeutic delivery systems. The LNP safety of Moderna, Acuitas, and Alnylam lipids, all of which are used in humans, was enhanced by introducing ester linkages, which may breakdown in water, to form ionizable lipids [201]. The RNA-based delivery technique must be chemically simple to enable human-scale production; for instance, let us consider a clinical trial setting where injecting a vaccine into a patient weighing 100 kg was administered with 6 mg/kg lipid and 0.3 mg/kg RNA (that is, a lipid:RNA mass ratio of 20:1), roughly 1 g of lipid would be needed for a single vaccine unit, assuming that there is no loss of lipid during formulation development. GalNAc conjugates, attached to siRNAs or ASOs independently, can be manufactured on a human scale (with the capacity to manufacture a large batch that complies with cGMP or Current Good Manufacturing Practice). Only LNPs with four lipid components and no targeting ligands were approved by regulators. It is essential that those developing therapeutic nanoparticles should know the benefits and drawbacks of incorporating targeted ligands [202]. Third, there needs to be a good on-target to off-target delivery ratio using the RNA delivery system. Biodistribution and function are two metrics that may be used to assess on- and off-target delivery. Biodistribution is required for effective cytoplasmic RNA delivery, but it is not adequate because endosomes retain more than 95% of RNA [203]. Additionally, studies have demonstrated that very low siRNA copy numbers are effective for gene knockdown in vitro [204], suggesting that siRNA functions catalytically. Fourthly, the dose of RNA that is effective must be far smaller than the dose that causes harm. Due to undetermined factors, mice have not shown to be reliable models of RNA toxicity; therefore, this can be seen in non-human primates. Fifth, even after shipping, the drug’s efficacy must be uniform throughout the batches. Work is being conducted to stabilise mRNA-based drugs for this reason. Storage of lyophilized CureVac mRNA at 40 °C for up to 6 months at room temperature maintained for 3 years showed no loss of functionality [205]. Furthermore, unless cryoprotectants such as sucrose are utilised, lyophilization may reduce stability for LNP- encapsulated mRNA by encouraging lipid crystallisation [206]. With the addition of 10% sucrose, for instance, RNA-complexed lipid nanoparticles may be lyophilized and kept for up to ≥ 8 months without refrigeration [207]. 

Sixth, in most clinical contexts, re-dosing the RNA drug so as to not compromise with the efficacy or safety is of outmost importance in maintaining the biological impact or “dose to effect.” Patients can safely be re-dosed with siRNA drugs when doses are spaced out by 3 weeks [108].

There has been successful double-dosing of mRNA vaccines, with either a 3-week or 4-weeks gap [200,208]. This impact is produced by a subpopulation of B lymphocytes, and it has been demonstrated that weekly re-dosing of mRNA reduces its efficiency in mice [209].

## 7. Clinical Angle

The potential exists to create new therapeutic approaches for CVD by targeting noncoding RNAs. While there have been obstacles in the past to the development of ASO and siRNAs delivery, recently conducted clinical trials reveal that chemical modifications along with targeting approaches have boosted efficiency, while at the same time they have decreased risk associated with undesired adverse effects. ORION-1 is a phase 2, multi-centric, double-blind, placebo-controlled, dose-escalation trial that recently reported its results on lowering LDL (low-density lipoprotein) level through the uptake of long-acting siRNA inclisiran, a subcutaneous vaccine, or PCSK9 into hepatocytes, facilitated due to its conjugation with triantennary N-acetylgalactosamine carbohydrates, which has high binding affinity for the ASGPRs in the liver. Phosphorothioate, 2′-O-methyl, and 2-fluoro nucleotide modifications are added to the siRNA to augment its stability as a molecule. With 2300 mg doses of inclisiran, PCSK9 and LDL cholesterol were significantly reduced and remained down for at least 240 days. Both the placebo and therapy groups experienced a comparable percentage of mild and severe adverse events (76% in both cases) [210]. Immune activation symptoms, a common worry associated with RNA therapies, were uncommon, and platelet counts were unaffected. Three patients treated with inclisiran exhibited transient increases in hepatic enzyme levels. A total of 4% and 7% of patients receiving a single dose and 2 doses of inclisiran experienced injection-site responses. These results are in line with those reported with monoclonal antibody therapy targeting PCSK9, but significantly different from those seen with earlier ASO therapies (wherein 80% of patients reported injection-site reaction) (e.g., trial results for ODYSEE showed a 5.9% success rate (To examine if it could minimise the risk associated with cardiovascular events in individuals experiencing an acute coronary syndrome; a randomized, double-blind, placebo-controlled, parallel-group study was conducted)) [211]. When compared with the 80% found in past ASO therapies’ injection-site studies, the lower figure is not surprising (e.g., 76% with mipomersen) [212]. However, the study did find that newer generations of siRNAs were substantially more effective and safe, and this was true even if this siRNA did not target a microRNA or long non-coding RNA in the cardiovascular system. To date, only one clinical study has been published demonstrating the safety and efficacy of LNA-based antimiRs (mirvarsen) for liver miR-122 targeting. Renal impairment due to Alport syndrome (kidney fibrosis) is being evaluated in another clinical trial using an antimiR against miR-21 (RG-012). In a multiple-dose phase 1 study, the concerned test substance was assessed in patients as well as healthy volunteers for a phase 2 placebo-controlled study (HERA study (Herceptin Adjuvant); NCT02855268) [182]. A list of vaccines that have reached various phases of clinical trials are listed in Table 3. Though few noncoding vaccines have reached clinical trial and eventually the market, some guidelines exist that suggest patients can extract benefit from such translational therapy. To begin with, patients with high priority disease, where molecular target cannot be addressed using conventional therapies, are to be considered. A large population of patients with severe clinical phenotypes may qualify for this therapy. Factors such as patients being unresponsive to conventional therapy, or the therapy itself being not efficient enough, or the therapy involves invasiveness, may be considered. CVD patients with high-priority diseases should have a unique pathomechanism at the centre of the condition, e.g., PCSK9 implicated in atherosclerosis and Ca^2+^ in heart failure. This kind of therapy can only be initiated in CVD patients with no irreversible damage such as myocardial fibrosis, myocardial infarction, and destruction of matrix.

## 8. Challenges Associated with Long Non-Coding RNA Therapeutics

As gene therapy targeting epigenetic modulators is still a relatively new idea and method compared with typical pharmacological targets and proteins, there are still some concerns regarding its potential side effects. The most significant concern is the lack of fundamental research on the function of miRNAs, lncRNAs, and their possible downstream consequences; as a result, the clinical usage of lncRNA-targeted medicines may result in unforeseen hazards and inappropriate pathological effects. In particular, off-target impacts may result in negative side effects. Therefore, highly specialised targeting strategies and delivery methods must be enhanced to ensure that the targeted epigenetic regulators are impacted. Highly cardio-specific targeted drug delivery methods pose a significant obstacle to the therapeutic application of lncRNAs. Another research suggested that in vitro differentiation of MSCs (mesenchymal stem cells) is inhibited by HOTAIR, especially differentiation toward adipogenic lineage. Despite poor sequence conservation, lncRNA possesses great tissue specificity. RNA-seq and unsupervised cluster analysis reveal that most lncRNAs exhibit tissue-specific expression. Furthermore, the effectiveness and safety of lncRNA medicines in humans remain uncertain because of a lack of significant evidence from clinical studies [225,226,227].

## 9. Conclusions

Important recent developments and therapeutic prospects of ncRNA research were addressed, with a focus on the translation of findings from fundamental science and in vivo/in vitro research into clinically useful new diagnostics and therapies. Meticulous patient selection turned out to be an important component in the effectiveness of clinical trials using ncRNAs as targets (miRs) and those using ncRNAs as tools (siRNAs) for therapeutic outcome. Consequently, given the rarity of validation of claimed outcomes, clinical trials conducted at large-scale are necessary for appraising the potential of ncRNAs as therapeutic agents in clinical contexts. Characterized selection criteria and adequate clinical outcome parameters, as well as carefully defined patient cohorts in which one molecular mechanism is responsible for pathogenesis and the main reason behind the onset and progression of any CVD, needs to be given careful thought for potential future clinical applications. Patients who are more likely to benefit from the work of ncRNA therapeutic approaches will be the primary focus for the success of future clinical trials [228].

The lncRNA is still at in its early stages with regard to how it acts at the molecular level in the body. Understanding the interaction between the delivery vehicles and RNA payload is the need of the hour. The interaction between these may affect tolerability and cellular targeting which needs attention and exploration. There is reported evidence that with change in the RNA payload, the nanoparticle movement changes. This is basically caused by the modification of both the nanoparticle and biomolecules as well as their interactions. Changes made in the RNA payload can affect the tropism associated with nanoparticles [141]. Moreover, it is the intrinsic properties of a cell that generate exogenous mRNA, which might not be suitable enough to produce SiRNA-mediated silencing of mRNA [229]. The chemical modifications made to ASOs and SiRNA have been found to be very effective in comparison with the unmodified ones [230]. The cell-specific activity can be tailored by modifying the untranslated portion of the mRNA [231,232].

This report suggests that RNA payloads can further be explored and improved for better results and to be effective enough to reach target patients. Another issue which we found during the review was that the extrapolation of tolerability and therapeutic effectiveness in rats and mice (small animal models) to humans and non-human primates requires appropriate study. There are stringent ethical issues associated with exploring drug delivery systems in non-human primates. Therefore, the development of appropriate small-animal-based models is suggested and the results can be scientifically translated to non-human primates. 

## Figures and Tables

**Table 1:** Illustrations of Some of the Most Important microRNAs in the CVD.

Sr No	Mi RNAs	Disease Implicated in	Model	Mechanisms	Ref.
1	miR-15	MI	Injury caused by ischemia/reperfusion in pigs and mice	Members of the microRNA-15 family have been shown to regulate mitochondrial activity by targeting proteins such as pyruvate dehydrogenase lipoamide kinase isozyme 4 and checkpoint kinase 1.	[10,11]
2	miR-24	MI	MI mouse model	The miR-24 family member Bcl2 is repressed. One characteristic of miRNA-mediated regulation of critical cellular events is exemplified by the BIM protein. It has been found that miR-24 inhibits apoptosis in cardiomyocytes in a cell-independent manner.	[12]
3	miR-34	MI	Mice	Through its regulation of suppression of the target gene PNUTS (protein phosphatase 1 nuclear-targeting subunit), miR-34a promotes telomere degradation and, by extension, age-related induction of cardiomyocyte cell death.	[13,14,15]
4	miR-92a	MI		Inhibits angiogenesis by reducing integrin α5 and sirtuin 1 expression.	[16,17,18]
5	miR-199a	MI	Pig model	Improves cardiac function by stimulating endogenous myocardial repair mechanisms.	[19]
6	miR-320	MI	Ischemia/reperfusion injury	Increased miR-320-3p expression and decreased Akt3 expression were seen in cardiomyocytes after H/R damage.	[20]
7	miR-590	MI	Patient	Regulating SOX4 can help restore the cell cycle process, which in turn boosts cardiomyocyte proliferation and reduces the severity of AMI.	[21]
8	miR-29	Cardiac fibrosis	Adult male and female C57BL/6 mice	Several matrix proteins are targeted by overexpression to reduce fibrosis.	[22]
9	miR-21	Cardiac fibrosis	Mice	Reduces the function of Spry1 (sprouty homologue 1), hence promoting the profibrotic ERK-MAP kinase signalling pathway.	[23]
10	miR-1,	Hypertrophy and heart failure	Male Sprague–Dawley rats	Its expression went down by a lot, as did the amount of collagen in the body and the activity of key profibrotic factors can also be involved in regulating fibrosis by going after Fbln2, a secreted ECM protein that plays a crucial part in tissue remodelling in diseased condition.	[24]
11	miR-133,	Hypertrophy and heart failure		When miR-133a is misregulated, it inhibits the expression of NFATc4, a mediator of hypertrophy.	[25]
12	miR-208,	Hypertrophy and heart failure	Mice	Overexpression of miR-208 inhibits the muscle-wasting proteins THAP1 and myostatin.	[26]
13	miR-25	Hypertrophy and heart failure	TAC model	Blocking microRNA-25a, cardiac function was restored by inhibiting the calcium uptake pump SERCA2a (sarco/endoplasmic reticulum Ca^2+^-ATPase 2a), which improved calcium management.	[27]
14	miR-212/132	Hypertrophy and heart failure	TAC mouse model	Inhibits the GFP/SERCA2a-3′-UTR expression.	[28]
15	miR-92a	Atherosclerosis and vascular remodelling	Mice	Inhibiting miR-92a, which functions as a proinflammatory regulator in endothelial cells by activating inflammatory cytokines and chemokines and augmenting monocyte adhesion, protects against endothelial dysfunction.	[29]
16	miR-126	Atherosclerosis and vascular remodelling	Female C57/BL6 mice	Through an indirect pathway mediated by apoptosis and VCAM-1 expression, suppression of stromal cell-derived factor-1/CXCL12 expression may attenuate leukocyte homing from blood circulation through the endothelium in vivo.	[30]
17	miR-146	Atherosclerosis and vascular remodelling	Patients with aortic valve stenosis	Downstream TLR4 signalling was controlled by a negative-feedback loop that included IL-1 receptor associated kinase 1 (IRAK1) and TNF-receptor associated factor 6 (TRAF6).	[31]
18	miR-181	Atherosclerosis and vascular remodelling	ApoE^−/−^ mice	IB kinase (IKK) complex regulatory/scaffold subunits TAB2 and NEMO are critical for inflammation-induced canonical NF-κB activation, which leads to the phosphorylation and degradation of IBs and the nuclear translocation of p65, thereby inhibiting vascular inflammation.	[32]

**Table 2:** Long non-coding RNA.

Sr No	lnc RNAs	Disease Implicated in	Model	Mechanisms	Ref.
1	Malat-1	MI	Postinfarct myocardium mice model	Post-MI, induced endothelial cell proliferation and ischemia-induced revascularization promoted by miR-145 regulation of TGF-1 expression cause cardiac fibrosis and decrease cardiac function.	[58,63]
2	lincRNA-p21	Atherosclerosis	Carotid artery injury model	Modulator of cell death by inhibiting p53 transcription during atherosclerosis is inhibited by lentivirus-mediated siRNA release targeting lincRNA-p21, causing neointima hyperplasia.	[60]
3	MIAT	MI	Mice	A molecular sponge for miR-15097 and a target gene modulator for the fibrosis-related factors miR-24, furin, and TGF-β1.	[58,64]
4	CARL	MI	Rat	Block particular microRNAs to control cardiomyocyte cell death.	[65]
5	CHRF	Cardiac hypertrophy	Transgenic mice that overexpress miR-489 in the heart	Directly bind miR-489 and control expression of MyD88 and hypertrophy.	[66]
6	Chast	Cardiac hypertrophy	TAC-operated mice	Repression of the pleckstrin homology domain containing protein family M member 1 promotes hypertrophy and prevents autophagy in cardiomyocytes.	[67]
7	Mhrt	Cardiac hypertrophy	TAC-operated mice	Mhrt activity is inhibited under pathological stress situations including pressure overload–induced hypertrophy and interferes with chromatin remodelling factor Brg1, regulating its target genes Myh6, Myh7, and osteopontin.	[68,69]
8	Meg3	Cardiac hypertrophy	C57BL6 mice	MMP-2 upregulation in cardiac fibroblasts induced cardiac fibrosis and diastolic dysfunction.	[59,70].
9	lncRNA H19	Coronary artery disease	Mice	Induced through vascular damage and human atherosclerotic plaques.	[71]
10	linc00323-003	Atherosclerosis		Inhibit the transcription of GATA2 (GATA-binding protein 2), a critical endothelial transcription factor that may control cell sproliferation and tube formation.	[72]

**Table 3:** Enlisted vaccines in clinical trials.

Vaccines	Type		Clinical Trial	Ref
TQJ230 (AKCEA-APO(a)-LRx),	Lipoprotein mRNA-specific ASO	Lowering of lipoprotein	Phase II	[181]
Volanesorsen	ASO	This ASO, which can be given subcutaneously, inhibits APOC3 mRNA stability by binding to it. Apoc-II was reduced by 80%, triglycerides by 71%, and HDL-C by 46%.	Phase II	[213]
CDR 132L	ASO	Heart failure	Phase I	[214]
MRG-110	ASO	Heart failure and ischemia; wounds (NDR)	Phase I (no development reported) (NDR)	[215]
SPC 4955	ASO	Hypercholesterolemia	Discontinued	[215]
SPC 5001	ASO	Hypercholesterolemia	Discontinued	[216]
ISIS CRPRx	ASO	Atrial fibrillation	Discontinued	[217]
ARO APOC3	siRNA	Dyslipidemias; hypertriglyceridemia (II); hyperlipoproteinemia type I (III)	Phase III; phase II	[218]
Zilebesiran	siRNA	Hypertension (II); preeclampsia (NDR)	Phase II; no development reported	[219]
Olpasiran	siRNA	CVD	Phase II	[220]
LY 3,561,774	siRNA	CVD; dyslipidaemias; metabolic disorders	Phase I	[221]
LY 3,819,469	siRNA	CVD; metabolic disorders	Phase I	[222]
SLN 360	siRNA	CVD (Pre); dyslipidaemias; hyperlipidaemia(I)	Preclinical; phase I	[223]
miR132-3p-inhibitor (CDR132L)	miR	Stable heart failure	Phase I	[224]

## Data Availability

Not applicable.

## Abbreviations

RNA: ribonucleic acid; lncRNA, long non-coding RNA; shRNA, short hairpin RNA; miRNA, micro RNA; mRNA, messenger RNA; DGCR, DiGeorge syndrome chromosomal region; ASO, antisense oligonucleotides; siRNA, small interfering RNA; LNP, lipid nanoparticle; AMI, acute myocardial infarction; DNA, deoxyribonucleic acid; ERK-MAP kinase, extracellular signal-regulated kinase-mitogen-activated protein kinase; TAC, transverse aortic constriction; SERCA2a, sarco/endoplasmic reticulum Ca^2+^-ATPase 2a; Hand2, heart- and neural crest derivatives-expressed protein 2; MHC, myosin heavy chain; PTEN, phosphatase and TENsin homolog deleted on chromosome 10; SREBF, sterol regulatory element binding proteins; HDL, high-density lipoprotein; Dlk1, delta-Like 1 homolog; NF-κB, nuclear factor-kappa B; TRAF6, tumour necrosis factor receptor-associated factor 6; LNA, locked nucleic acid; ESCRT-I, endosomal sorting complexes required for transport I; TSG101, tumour susceptibility gene 101 protein; MIAT, myocardial infarction-associated transcript; TGF-1, transforming growth factor-1; MIAT, myocardial infarction-associated transcript; CARL, cardiac apoptosis-related lncRNA; MDRL, mitochondrial dynamic-related lncRNA; APF, autophagy-promoting factor; CHRF, cardiac hypertrophy-related factor; Mhrt, myosin heavy chain-associated RNA transcript; ANRIL, antisense noncoding RNA in the INK4 (inhibitor of CDK4) locus; GATA2, GATA-binding factor 2; PCSK9, proprotein convertase subtilisin/kexin type 9; Malat-1, metastasis-associated lung adenocarcinoma transcript 1; CRISPR/Cas9, clustered regularly interspaced short palindromic repeats/clustered regularly interspaced short palindromic repeat-associated 9; PHB2, prohibitin 2; CUPID, calcium upregulation by percutaneous administration of gene therapy in cardiac disease; scFvs, single-chain variable fragments; DARPins, designed ankyrin repeat proteins.
